# Genotype Change in Circulating JEV Strains in Fujian Province, China

**DOI:** 10.3390/v15091822

**Published:** 2023-08-26

**Authors:** Nihua Dong, Xinya Zhang, Hailong Zhang, Jiayang Zheng, Yafeng Qiu, Zongjie Li, Beibei Li, Ke Liu, Donghua Shao, Zhiyong Ma, Jianchao Wei

**Affiliations:** Shanghai Veterinary Research Institute, Chinese Academy of Agricultural Science, Shanghai 200241, China; dongnihua0519@163.com (N.D.); 13770510390@163.com (X.Z.); zhanghailong1997@163.com (H.Z.); asd126163@163.com (J.Z.); yafengq@shvri.ac.cn (Y.Q.); lizongjie@shvri.ac.cn (Z.L.); lbb@shvri.ac.cn (B.L.); liuke@shvri.ac.cn (K.L.); shaodonghua@shvri.ac.cn (D.S.)

**Keywords:** JEV, Fujian, mosquito

## Abstract

Japanese encephalitis (JE), found in pigs, is a serious mosquito-borne zoonotic infectious disease caused by the Japanese encephalitis virus (JEV). JEV is maintained in an enzootic cycle between mosquitoes and amplifying vertebrate hosts, mainly pigs and wading birds. It is transmitted to humans through the bite of an infected mosquito, allowing the pathogen to spread and cause disease epidemics. However, there is little research on JEV genotype variation in mosquitoes and pigs in Fujian province. Previous studies have shown that the main epidemic strain of JEV in Fujian Province is genotype III. In this study, a survey of mosquito species diversity in pig farms and molecular evolutionary analyses of JEV were conducted in Fujian, China, in the summer of 2019. A total of 19,177 mosquitoes were collected at four sites by UV trap. Four genera were identified, of which the *Culex tritaeniorhynchus* was the most common mosquito species, accounting for 76.4% of the total (14,651/19,177). *Anopheles sinensi* (19.25%, 3691/19,177) was the second largest species. High mosquito infection rateswere an important factor in the outbreak. The captured mosquito samples were milled and screened with JEV-specific primers. Five viruses were isolated, FJ1901, FJ1902, FJ1903, FJ1904, and FJ1905. Genetic affinity was determined by analyzing the envelope (E) gene variants. The results showed that they are JEV gene type I and most closely related to the strains SH-53 and SD0810. In this study, it was found through genetic evolution analysis that the main epidemic strain of JE in pig farms changed from gene type III to gene type I. Compared with the SH-53 and SD0810 strains, we found no change in key sites related to antigenic activity and neurovirulence of JEV in Fujian JEV and pig mosquito strains, respectively. The results of the study provide basic data for analyzing the genotypic shift of JEV in Fujian Province and support the prevention and control of JEV.

## 1. Introduction

Japanese encephalitis virus (JEV), a member of the genus Flavivirus, is one of the smallest viruses in the Flaviviridae. The JEV virion is spherical, with an icosahedral symmetry of approximately 40 to 50 nm in diameter, and has a single-stranded RNA molecule of approximately 11 kb, making it a positive-stranded RNA virus with an envelope. The Flavivirus genome contains a single open reading frame (ORF) that encodes a polyprotein. This polyprotein encodes three structural proteins, which are encoded in the 5′ third of the ORF sequences: the capsid (C), pre-membrane/membrane (prM/M), and envelope (E) proteins. Seven non-structural (NS) proteins (NS1, NS2A, NS2B, NS3, NS4A, NS4B, and NS5) are encoded in the remaining 3′ two-thirds sequences. The E protein is considered to be the most important immunogen [1,2,3,4,5]. JEV can cause serious infectious disease symptoms in humans and spread worldwide [6]. Many animals infected with JEV can become the source of transmission, of which pigs are the important host, the most important source of infection, and the amplifying host of the disease. JEV circulates in “mosquito-pig-mosquito” and “porcine-mosquito-human” cycles [7,8,9]. JEV can cause encephalitis in piglets, retention heat in fattening pigs, sow abortion, and male orchitis in pigs, which has caused huge economic losses to the pig industry [1]. JEV can cause irreversible damage in infected humans, manifesting symptoms such as high fever, impaired consciousness, convulsions, and even leading to mental retardation, aphasia, and ankylosis of hands and feet [3,10,11].

The pathogen was first found in Japan in 1934, and JEV was also isolated in China in 1939. At present, the disease is prevalent throughout Asia, mainly in tropical, subtropical, and temperate countries in eastern Asia, of which Asia includes China, Japan, India, Malaysia, Vietnam, and other countries as the main epidemic areas of JE [12,13,14]. In the tropical and subtropical regions of Asia, the virus is mainly transmitted through *Culex* mosquitoes [8,15]. JEV’s high mortality and disability rates make it a public health priority in Asian countries. The spread of JEV is highly dynamic; its spread and outbreak patterns are affected by fluctuating environmental and social factors [4,16,17]. Clinically, different hosts infected with JEV exhibit different clinical symptoms. Most human infections are asymptomatic, but children and the elderly may develop mild infections, which in severe cases lead to encephalitis. The incubation period of the disease is 4–15 days, and patients with JE may show nonspecific initial symptoms such as fever, headache, nausea, diarrhea, vomiting, and myalgia. These may be followed by acute encephalitis with neurological symptoms [18]. Pigs are one of the most susceptible animals to JEV, and pigs can show typical clinical signs after infection with JEV [19,20]. Piglets infected with JEV show obvious signs of encephalitis, pregnant sows infected with JEV develop abortions and stillbirths, and testicular inflammation occurs in boars [21]. Pathological changes caused by JEV are mainly manifested in tissues and organs such as the brain, spinal cord, testes, and uterus, where obvious meningeal and cerebral vascular congestion, hemorrhage, and edema, as well as substantial foci of testicular congestion and necrosis, can be seen [22]. Aborted fetuses may commonly present with cerebral edema, increased ascites with subcutaneous hemorrhagic infiltration, and varying fetal size, with some presenting as mummified fetuses. At present, there is no specific drug for the treatment of JE, prevention is the mainstay of the breeding industry, and the treatment of JE is not advocated. However, disease prevention for humans and livestock can be achieved through health measures and vaccination. In addition, as a natural focus disease transmitted by mosquitoes, the incidence of JE will be affected by climate factors such as temperature and rainfall [2,23,24,25].

Previous research on JEV has mostly focused on human sources, and relatively few studies have been conducted on mosquito-derived viruses and pig-derived JEVs. This paper can grasp the changes in different subtypes in Fujian. Therefore, research on the local pigpens seasonal population of mosquitoes, the JEV genotype, and natural JEV transmission is important for understanding the transmission and spread of JEV. Fujian has a subtropical monsoon climate. Influenced by monsoon circulation and topography, it is warm and humid with abundant rainfall. In history, Fujian was a province with a high incidence of JE. After the comprehensive use of the JE vaccine in the 1980s, the epidemic was brought under control [26]. However, vaccines are expensive and require multiple doses to maintain efficacy and immunity. Although Japanese encephalitis (JE) has been decreasing in severity in mainland China, it remains a serious swine infectious disease. Therefore, monitoring and controlling major mosquito vectors is a more promising strategy to reduce transmission, and it is very important to establish continuous and effective detection and early warning mechanisms. On the one hand, we need to monitor the molecular genetic variation characteristics of JEV to prevent new epidemics of JEV. On the other hand, it is necessary to monitor mosquito vectors in pig farms. JEV has the characteristic of insect vector transmission, especially in the mosquito breeding season. In order to further grasp the basic background of JEV in pig farms in Fujian Province, this study carried out nucleic acid detection of JEV from mosquito samples collected at four monitoring points in Fujian Province from July to September 2019 and determined the molecular characteristics of JEV strains in pig farms in Fujian Province through the E gene sequence analysis.

## 2. Materials and Methods

### 2.1. Mosquito and Swine Blood Sample Collection

Mosquitoes were trapped daily using black ultraviolet (UV)-light traps (12 V, 300 mA; Photocatalytic Technology Mosquito Catcher Device, Electrical Technology, Guangdong, China) at Fuqing (FQ)-YC, Sanming (SM)-YS, Nanping (NP)-YR, and Nanping(NP)-LTS (Figure S1, Table S1) in Fujian Province from late July to early September (local mosquito activity season) in 2019. Six black UV-light traps were set for each farm: three placed in different barns and three under the eaves outside [27]. The collected mosquitoes were frozen at −20 °C and male mosquitoes were removed from the samples. The specimens were classified and divided according to different mosquito species. Fifty mosquitoes were pooled, labeled, and stored in liquid nitrogen. A total of 120 sera from pigs at 3 months of age were collected from Fuqing-YC, Sanming-YS, Nanping (NP)-YR, and Nanping-LTS, and 30 serum samples were obtained from each farm.

### 2.2. JEV Detection and Virus Isolation

After subdividing the collected mosquitoes for grinding, total RNA was extracted from the original biological samples and pig blood using Trizol (Takara Bio, Kusatsu, Japan). The cDNA libraries were prepared using the M-MLV (H-) Reverse Transcriptase kit (Vazyme Biotech Co., Ltd., Piscataway, NJ, USA) following the operating instructions. The JEV E gene sequence was amplified using JEV-E-F1 and JEV-E-R1 primers designed in this laboratory (Table 1). PCR-positive samples were sent to Sangon Biotech (Shanghai, China) Co., Ltd. (No. 698, Xiangmin Road, Songjiang District, Shanghai, China) for sequencing using JEV-E-F2 and JEV-E-R2.

Baby hamster kidney (BHK)-21 cells were cultured in 93% Dulbecco’s modified Eagle’s medium supplemented with 7% heat-inactivated fetal bovine serum (FBS; Invitrogen) and 100 U/mL of penicillin and streptomycin. The mosquito pools (50 mosquitoes/pool) were triturated in minimum essential medium (containing 1% FBS, 100 U/mL of penicillin, 100 U/mL of streptomycin, and lL/mL of fungizone) using the TissueLyser (Qiagen, Valencia, CA, USA) at room temperature for 3 min. The homogenates were clarified by centrifugation at 12,000 rpm and 4 °C for 10 min. Clarified homogenates (100 µL) were inoculated onto monolayers of BHK-21 cells for 1 h at 37 °C, respectively. Then, the cells were washed with minimum essential medium and were maintained at 37 °C adding the minimum essential medium containing 5% FBS, 100 U/mL of penicillin, 100 lg/mL of streptomycin, and 1 lL/mL of fungizone [28]. Cells were observed daily for cytopathic effects (CPEs) from days 1–7 postinfection. All experiments for the virus isolation were performed in a biosafety level 2 cell culture laboratory established at Shanghai Veterinary Research Institute, China.

### 2.3. JEV E Gene Sequence Analysis

The nucleotide sequence of the E gene of a classical JEV strain was searched from GenBank. The evolutionary relationships of E genes were compared with 45 references of JEV in GenBank from different decades, countries, and hosts (Table 2). Multiple sequence alignments and sequence similarity calculations between aligned nucleotide and amino acid sequences were performed using DNASTAR software (Madison, WI, USA) [29]. Multiple sequence alignments and phylogenetic trees were produced using MEGA 6.0 software and constructed from aligned nucleotide sequences using the neighbor-joining method. The stability of the tree obtained was established by bootstrapping analysis with 1000 replications [30].

Minimum infection rate (MIR): The MIR (number of positive pools/total specimens tested ×1000) was calculated for each mosquito species and virus collected over the duration of the project. The MIR is expressed as the number of positive mosquitoes per 1000 tested and assumes that a positive pool contains only 1 infected mosquito.

## 3. Results

### 3.1. Identification of Mosquitoes

A total of 19,177 mosquito specimens representing seven species from four genera (14,651 *Culex tritaeniorhynchus* (76.40%), 38 *Culex bitaeniorhynchus* (0.2%), 3691 *Anopheles sinensis* (19.25%), 55 *Culex pipiens pallens* (0.29%), 645 *Aedes vexans* (3.36%), and 97 *Armigeres subbalbeatus* (0.51%)) were collected from July to September in 2019 (Table 3). *Culex mosquito* was the dominant species, with 14,744 mosquitoes, accounting for 76.89% of the total mosquito collection. The collected specimens were preserved at −20 °C.

### 3.2. JEV Detection and Gene Sequencing

Five E gene sequences obtained from the mosquitoes and pigs were 1500 nt long, respectively, and have been deposited in GenBank (acc. no. FJ1901, FJ1903, and FJ1905 for mosquito and FJ1902 and FJ1904 for pigs) (Table 4).

The 19,177 mosquitoes were sorted into 393 pools according to species, location, and date of collection. Most pools have 50 mosquitoes. Less than 50 mosquitoes also serve as a mosquito pool. In total, 6 out of 393 (1.5%) mosquito pools were PCR-positive. In addition, 8 of 120 (6%) swine blood samples were PCR-positive. All the PCR-positive samples were processed for virus isolation. A total of three isolates, FJ1901, FJ1903, and FJ1905, were obtained from mosquito samples, resulting in CPEs (Table 5). Eight PCR-positive swine blood samples were also used for virus isolation, resulting in two isolates, FJ1902 and FJ1904. Isolates FJ1901 and FJ1902 caused CPE in 2 days. CPE caused by FJ1903, FJ1904, and FJ1905 isolates began 4 days after inoculation.

### 3.3. Molecular Characterization and Phylogenetic Analysis Based on the E Genes of JEV

**Figure 1:**
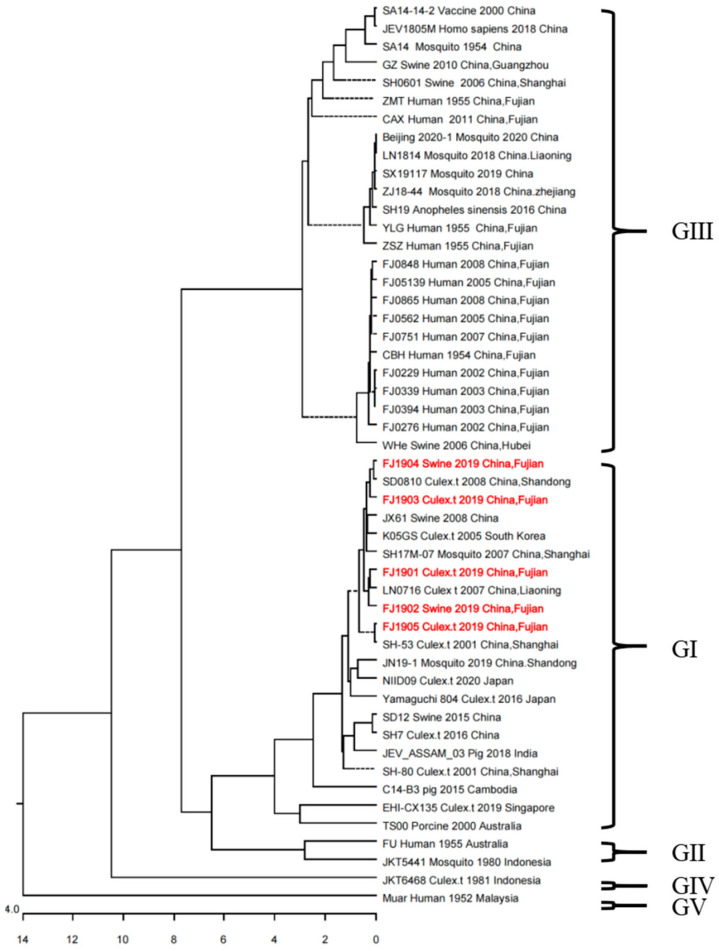
Phylogenetic tree of relationships between JEV strains based on E gene variation. Full nucleotide sequences of different JEV E genes were aligned, and evolutionary analyses were conducted in MEGA 6.0 using 1000 bootstrap replicates of the sequence data. Five viruses were isolated, FJ1901, FJ1902, FJ1903, FJ1904, and FJ1905 (indicated in red).

We conducted PCR using JEV-E-F2 and JEV-E-R2 primers (Table 1) to amplify the E gene of JEV from the FJ1901, FJ1902, FJ1903, FJ1904, and FJ1905 isolates and constructed a phylogenetic tree based on the sequences of the PCR products. We used 45 representative JEV strains to perform multiple sequence alignments and phylogenetic analyses (Table 2). We compared nucleotide sequences of the E gene. The homology of the Fujian 1901, Fujian 1902, Fujian 1903, Fujian 1904, and Fujian 1905 isolates, compared with the genotype I JEV strain, was 98.2–99.6%, respectively, which was higher than that for the other genotypes (88.5–88.8% and 83.1–87.4%). The Fujian isolates showed the highest homology with the classical JEV genotype I SH-53 and JEV genotype I SD0810. Phylogenetic analysis of the JEV E fragments showed that all Fujian JEV isolates were genotype I (Figure 1). The cladogram showed that isolates FJ1901 and FJ1902 were closely related to strain LN0716, strains FJ1903 and FJ1904 were closely related to strain SD0810, and strain FJ1905 was closely related to strain SH-53 (Figure 1). Our results indicated that genotype I was the major JEV subtype circulating in Fujian. In addition to the Chinese strains, we included the latest genotype I JEV strains isolated in China and Asian countries (Singapore, Japan, Korea, India, etc.) for phylogenetic analysis, and found that the newly isolated Fujian JEV strain was closely related to the latest isolates from other parts of China, as well as to the South Korean strain K05GS.

### 3.4. Analysis of Mutation at Critical Amino Acid Residues

Envelope protein E is a major structural protein consisting of 500 amino acids. The E protein plays an important role in JEV immunogenicity, cell fusion and infection, and viral maturation. We compared amino acid sequences of the Fujian JEV mosquito strain (FJ1901, FJ1903, and FJ1905) and pig strain (FJ1902 and FJ1904) with that of the live vaccine strain SA14-14-2 and other similar strains (Table 6). Five amino acid residues in the newly detected Fujian JEV strains differed from those in the live attenuated vaccine SA14-14-2-derived strain (SA14): E107 (Phe→Leu) E129 (Thr→Met) E138 (Lys→Glu) E176 (Val→Ile) E222 (Ala→Ser) E244 (Gly→Glu) E264 (His→Gln) E279 (Met→Lys) E315 (Val→Ala) E327 (Ser→Thr) E366 (Alal→Ser) E439 (Arg→Lys). However, some of the key sites E138, E47, E176, E123, E244, and E107 [31,32,33,34,35,36,37], which have been shown to influence virulence and antigenic activity, were unchanged compared with other genotype I JEV strains.

MIR: The MIR of JEV in *Cx. Tritaeniorhynchus* was 0.41/1000 (Table 7).

## 4. Discussion

JE can occur all year round but mainly occurs in summer or early autumn with more rain [38,39,40]. JEV is active in most Chinese provinces, and nearly 50% of the world’s reported cases occur in China [41,42,43]. Fujian is located in the subtropical region, with a mild climate, abundant rainfall, and a high density of insect vectors, and has historically been a province with a high incidence of JE epidemics in China. The main strains isolated and identified in China are JEV genotypes I, III, and V [44]. In past years, there has been a mixed epidemic of genotype I and genotype III strains, but it is still dominated by genotype III [24]. In 2010, mosquito samples were collected in Fujian Province, mainly *Culex trinasis* and *Anopheles sinensis*, and JEV genotype I was isolated from the samples [26,45]. However, the Gene Bank shows that the strains isolated in Fujian Province are mainly genotype III JEV. Detection of JEV in mosquitoes and pigs around pigpens confirmed that there is a risk of JEV infection in this area. In this study, mosquito and pig blood samples were collected from four pig farms in three regions of Fujian, and the results showed that the mosquito species were mainly *Culex tritaeniorhynchus* and *Anopheles sinensis*, which is consistent with the previous results. According to the full sequence of the E protein gene, JEV is divided into five genotypes, and the E gene is considered to be well-represented for evolutionary analysis and has become the main analysis method in recent years [46]. Therefore, in this experiment, the isolated virus was sequenced in the full sequence of the E gene and genetic evolution analysis.

We found that E gene variations indicate that the Fujian JEV strain is of type I. The sequence identities between the Fujian JEV mosquito and pig strains of the E gene were 97.2% and 99.8%, respectively, confirming the local natural JEV transmission cycle within mosquitoes and pigs. We also analyzed mutations in critical amino acid residues. Researchers compared the nucleotide sequences of JE virus strains with different virulence to elucidate the level of genes affecting the virulence of JEV [31,32,33,34,35,36,37]. There are some amino acid site differences between different genotypes of JEV, and the virulence of the virus is often affected by these differences in amino acid sites. Compared with the classical JEV genotype I SH-53 and SD0810, the JEV strain isolated in this study did not show any mutation at the above key sites in the Fujian JEV strain.

Epidemiological surveillance and ecological reports show that in some Asian countries, such as China, Japan, South Korea, Malaysia, Thailand, etc., the epidemic trend of JEV has shown genotype conversion; that is, genotype I JEV has gradually replaced GIII type as the dominant genotype of the main epidemic. In recent years, researchers have reported the isolation of genotype I JEV from mosquito samples collected in Shanghai, Henan, Tibet, and other regions [47,48,49,50,51,52]. Therefore, it is necessary to further strengthen the molecular evolution analysis and JEV isolation and detection of pig farm mosquito vectors and JEV in the insect vector concentration sites in Fujian Province and pay close attention to the transformation of the JEV genotype. It provides a theoretical basis for the prevention and control of JE in Fujian Province. The main JEV vaccine strains in common use are Nakayama, Beijing-1, Beijing-3, and SA14-14-2, all of which are JEV genotype III strains, and these vaccines do not provide complete protection against GI [53,54,55]. A comparison of sequence data revealed that the Fujian JEV mosquito/pig strains and the SA-14-14-2 vaccine differed at amino acid sites; key regions that determine antigenic activity were different. This suggests that the Fujian JEV strain’s antigenicity is different from the SA-14-14-2 vaccine strain. In theory, the currently used JEV vaccine should be ineffective in protecting against infection by the Fujian JEV strain.

The epidemiological trend of JEV in Fujian Province has shifted from genotype III to genotype I. However, the molecular mechanism of genotype switching has not been clearly explained. Some current studies suggest that the cause of genotype conversion may be related to host adaptation [56]. Xiao et al. found a host adaptation advantage of JEV genotype I over JEV genotype III in amplified hosts, especially avian hosts [29]. At the same time, Han et al. showed that JEV genotype I is more adaptive within a given host range relative to JEV genotype III [57]. That may be an important cause of genotype switching in epidemics of JEV. We analyzed nucleotide sequence variations in the E gene, indicating a relatively high degree of homology between the Fujian JEV mosquito and pig strains. On the other hand, the key E gene sites of the Fujian strain are identical to other JEV genotype I strains, and since the persistence of Flaviviruses in insects and mammals implies that it cannot withstand many variations, which suggests that JEV is genetically relatively stable.

This study reported on the vector mosquito ecology and that genotype I is now considered the dominant strain in Fujian. Monitoring changes in JEV genotypes is important for developing effective control strategies. Therefore, it will require implementing long-term continuous monitoring of JEV-infected mosquitoes and pigs, recording genotypic distribution and genetic variation in JEV, establishing strategies to control mosquito and mosquito-borne diseases, timely assessment of the transmission potential of pigs as hosts, and providing data support for Japanese encephalitis control.

## Figures and Tables

**Table 1:** Primers used for RT-PCR assay to detect the E gene of JEV in mosquitoes.

Primers	Primer Sequence (5′—′)
JEV-E-F1	TTGGTCGCTCCGGCTTACA
JEV-E-R1	GGTTTTCCGAGGTAGTGGTTC
JEV-E-F2	TGCTGGTCGCTCCGGCTTA
JEV-E-R2	GATGTCAATGGCACATCCAGT

**Table 2:** Background information on 45 JEV strains compared with Fujian JEV isolates in this study.

Strain	Date	Region	Host	GenBank
FJ1901 ^(1)^	2019	China, Fujian	*Culex tritaeniorhynchus*	OQ181396
FJ1902 ^(1)^	2019	China, Fujian	Swine	OQ181397
FJ1903 ^(1)^	2019	China, Fujian	*Culex tritaeniorhynchus*	OQ181398
FJ1904 ^(1)^	2019	China, Fujian	Swine	OQ181399
FJ1905 ^(1)^	2019	China, Fujian	*Culex tritaeniorhynchus*	OQ181400
FJ08-48	2008	China, Fujian	Human blood	GQ856663
FJ05-139	2005	China, Fujian	Human blood	GQ856661
FJ05-62	2005	China, Fujian	Human blood	GQ856660
FJ07-51	2007	China, Fujian	Human blood	GQ856662
FJ08-65	2008	China, Fujian	Human	GQ856664
CBH	1954	China, Fujian	Human blood	JN381860
FJ0229	2002	China, Fujian	Human blood	JF706273
FJ0394	2003	China, Fujian	Human blood	JN381858
FJ0339	2003	China, Fujian	Human blood	JN381859
FJ0276	2002	China, Fujian	Human blood	JN381867
Whe	2006	China	Swine	EF107523
SA 14-14-2	2000	China, Beijing	-	AF315119
SA14	1954	China	Mosquito	U14163
Gz	2010	China	Swine	KC915016
SH0601	2006	China	Swine	EF543861
ZMT	1955	China, Fujian	CSF	JN706283
CAX	2011	China	CSF	JN381865
ZSZ	1955	China, Fujian	CSF	JN381862
YLG	1955	China, Fujian	CSF	JF706280
SD0810	2008	China, Shandong	*Culex tritaeniorhynchus*	JF706286
SH17M-07	2007	China	-	EU429297
SH-53	2001	China, Shanghai	*Culex tritaeniorhynchus*	JN381850
JX61	2008	China	Swine	GU556217
LN0716	2007	China, Liaoning	*Culex tritaeniorhynchus*	JN381849
SH-80	2001	China, Shanghai	*Culex tritaeniorhynchus*	JN381848
JKT5441	1980	Indonesia	Mosquito	JQ429306
FU	1995	Australia	Human blood	AF217620
JKT6468	1981	Indonesia	*Culex tritaeniorhynchus*	AY184212
Muar	1952	Malaysia	Human brain	HM596272
C14-B3	2015	Cambodia	Pig	KY927817
Yamaguchi 804	2016	Japan	*Culex tritaeniorhynchus*	LC461957
JEV_ASSAM_03	2018	India	Pig	MZ702743
JEV1805M	2018	China	Homo sapiens	MN639770
K05GS	2005	South Korea	*Culex tritaeniorhynchus*	KR908702
Beijing 2020-1	2020	China.Beijing	Mosquito	OP588746
NIID09	2020	Japan	*Culex tritaeniorhynchus*	LC623822
JN19-1	2019	China.Shandong	Mosquito	OM572538
LN1814	2018	China.Liaoning	Mosquito	OM572545
SX19117	2019	China	Mosquito	OM572540
ZJ18-44	2018	China.zhejiang	Mosquito	OM572550
TS00	2000	Australia	Porcine	MT253732
SH19	2016	China	*Anopheles sinensis*	MH753131
SH7	2016	China	*Culex tritaeniorhynchus*	MH753129
EHI-CX135	2019	Singapore	*Culex tritaeniorhynchus*	ON804798
SD12	2015	China	Swine	MH753127

^(1)^: The strain isolated from Fujian province in this study.

**Table 3:** Distribution of mosquitoes in pig farms in Fujian, China.

Species	Location				Total No. (%)
	Fuqing (FQ)	Sanming (SM)	Nanping (NP)		
	YC No. (%)	YS No. (%)	YR No. (%)	LTS No. (%)	
*Culex tritaeniorhynchus*	3954 (69.97%)	3647 (72.06%)	3501 (85%)	3549 (81.66%)	14,651 (76.40%)
*Culex bitaeniorhynchus*	11 (0.19%)	0 (0%)	8 (0.19%)	19 (0.44%)	38 (0.2%)
*Anopheles sinensis*	1107 (19.59%)	1328 (26.24%)	522 (12.67%)	734 (16.89%)	3691 (19.25%)
*Culex pipiens pallens*	7 (0.12%)	13 (0.26%)	26 (0.63%)	9 (0.21%)	55 (0.29%)
*Aedes vexans*	572 (10.12%)	73 (1.44%)	0 (0%)	0 (0%)	645 (3.36%)
*Armigeres obturbans*	0 (0%)	0 (0%)	62 (1.51%)	35 (0.81%)	97 (0.51%)
Total	5651	5061	4119	4346	19,177
Percent of the sites	(29.47%)	(26.39%)	(21.48%)	(22.66%)	(100%)

**Table 4:** Summary of JEV isolates from mosquitoes and pig serums collected in Fujian, China.

No.	Isolates	Collection Sites	Habitat	Host	Genotype
1	FJ1901	YC	pigpen	*Cx. tritaeniorhynchus*	GI
2	FJ1902	YC	pigpen	Swine	GI
3	FJ1903	YS	pigpen	*Cx. tritaeniorhynchus*	GI
4	FJ1904	YS	pigpen	Swine	GI
5	FJ1905	LTS	pigpen	*Cx. tritaeniorhynchus*	GI

**Table 5:** Classification of samples collected from pig farms in Fujian Province and isolation of JEV virus in positive samples.

Species	No. of Samples				No. of Positive Samples			
	YC	YS	YR	LTS	YC	YS	YR	LTS
*Culex tritaeniorhynchus*	79	73	70	71	2 (1 *)	1 (1 *)	1	2 (1 *)
*Culex bitaeniorhynchus*	1	0	1	1	0	0	0	0
*Anopheles sinensis*	23	27	11	15	0	0	0	0
*Culex pipiens pallens*	1	1	1	1	0	0	0	0
*Aedes vexans*	12	2	0	0	0	0	0	0
*Armigeres obturbans*	0	0	2	1	0	0	0	0
Swine blood	30	30	30	30	3 (1 *)	2 (1 *)	1	2

No. of samples: the number of samples of each mosquito and pig blood from the farm; no. of positive samples: the number of samples verified as positive by PCR; *: the number of JEVs isolated from positive samples.

**Table 6:** Comparison of key Japanese Encephalitis Virus E protein amino acid residues at sites associated with virulence in different Japanese Encephalitis Virus strains.

	JEV Strain GenBank		E47	E76	E107	E123	E129	E138	E160	E176	E222	E227	E244	E259	E264	E279	E312	E315	E327	E366	E408	E439	E441	E487
GII	SA14-14-2	AF315119	N	T	F	S	T	K	G	V	A	S	G	E	H	M	K	V	S	A	S	R	V	T
	P3	U47032	N	M	L	S	A	E	G	I	A	P	E	E	Q	K	K	A	S	A	L	K	V	T
	SH0601	EF543861	N	M	L	S	T	E	G	I	A	P	E	E	Q	K	K	A	S	A	L	K	V	T
	FJ0339	JN381859	N	T	L	S	T	E	R	I	A	P	E	E	Q	K	R	A	S	A	S	K	I	I
	Fj02-29	JF706273	N	T	L	S	T	E	R	I	A	P	E	E	Q	K	R	A	S	A	S	K	I	I
	FJ07-51	GQ856662	N	T	L	S	T	E	G	I	A	P	E	K	Q	K	R	A	S	A	S	K	I	I
	FJ05-62	GQ856660	N	T	L	S	T	E	G	I	A	P	E	K	Q	K	R	A	S	A	S	K	I	I
	FJ08-48	GQ856663	N	T	L	S	T	E	G	I	A	P	E	K	Q	K	R	A	S	A	S	K	I	I
GI	FJ1901	OQ181396	N	T	L	S	M	E	G	I	S	S	E	E	Q	K	K	A	T	S	S	K	V	T
	FJ1902	OQ181397	N	T	L	S	M	E	G	I	S	S	E	E	Q	K	K	A	T	S	S	K	V	T
	FJ1903	OQ181398	N	T	L	S	M	E	G	I	S	S	E	E	Q	K	K	A	T	S	S	K	V	T
	FJ1904	OQ181399	N	T	L	S	M	E	G	I	S	S	E	E	Q	K	K	A	T	S	S	K	V	T
	FJ1905	OQ181400	N	T	L	S	M	E	G	I	S	S	E	E	Q	K	K	A	T	S	S	K	V	T
	SH-53	JN381850	N	T	L	S	M	E	G	I	S	S	E	E	Q	K	K	A	T	S	S	K	V	T
	SH-80	JN381848	N	T	L	S	M	E	G	I	S	S	E	E	Q	K	K	A	T	S	S	K	V	T

**Table 7:** Minimum infection rate of JEV in mosquitoes in this study.

Species	Specimen	Positive Pool	MIR ^(1)^
*Culex tritaeniorhynchus*	14,651	6	0.41/1000

^(1)^: Minimum infection rate (MIR) expressed as number infected/1000 tested.

## Data Availability

All data are available in the main text or Supplementary Materials.

## Supplementary Materials

The following supporting information can be downloaded at: https://www.mdpi.com/article/10.3390/v15091822/s1. Table S1: Information on pig farms for mosquito and swine blood collection. Figure S1: Map of the sampling sites in Fujian Province, China.
