# Supplementary material for: Genotype Change in Circulating JEV Strains in Fujian Province, China
Source: Viruses. 2023 Aug 26;15(9):1822. doi: 10.3390/v15091822 (PMC10536422; doi:10.3390/v15091822)
Supplement: Supplementary file 1 [file viruses-15-01822-s001.zip › viruses-2551982-supplementary.pdf]

Supplementary Table S1. Information on pig farms from which samples were collected

County	Farm	Farm size	Aerage Temp.°C	Altitude (m)	Longitude	Latitude
Fuqing (FQ)	YC	>5000	19℃-23℃	7	119.563087	25.473633
Sanming (SM)	YS	>3000	14℃-19℃	387	117.437698	26.174683
Nanping (NP)	YR	>2000	16℃-20℃	212	118.7005	27.492241
Nanping (NP)	LTS	>2000	15℃-19℃	341	118.585342	28.122961
