# Supplementary figures and images for: Genotype Change in Circulating JEV Strains in Fujian Province, China
Source: Viruses. 2023 Aug 26;15(9):1822. doi: 10.3390/v15091822 (PMC10536422; doi:10.3390/v15091822)

## Slide 1
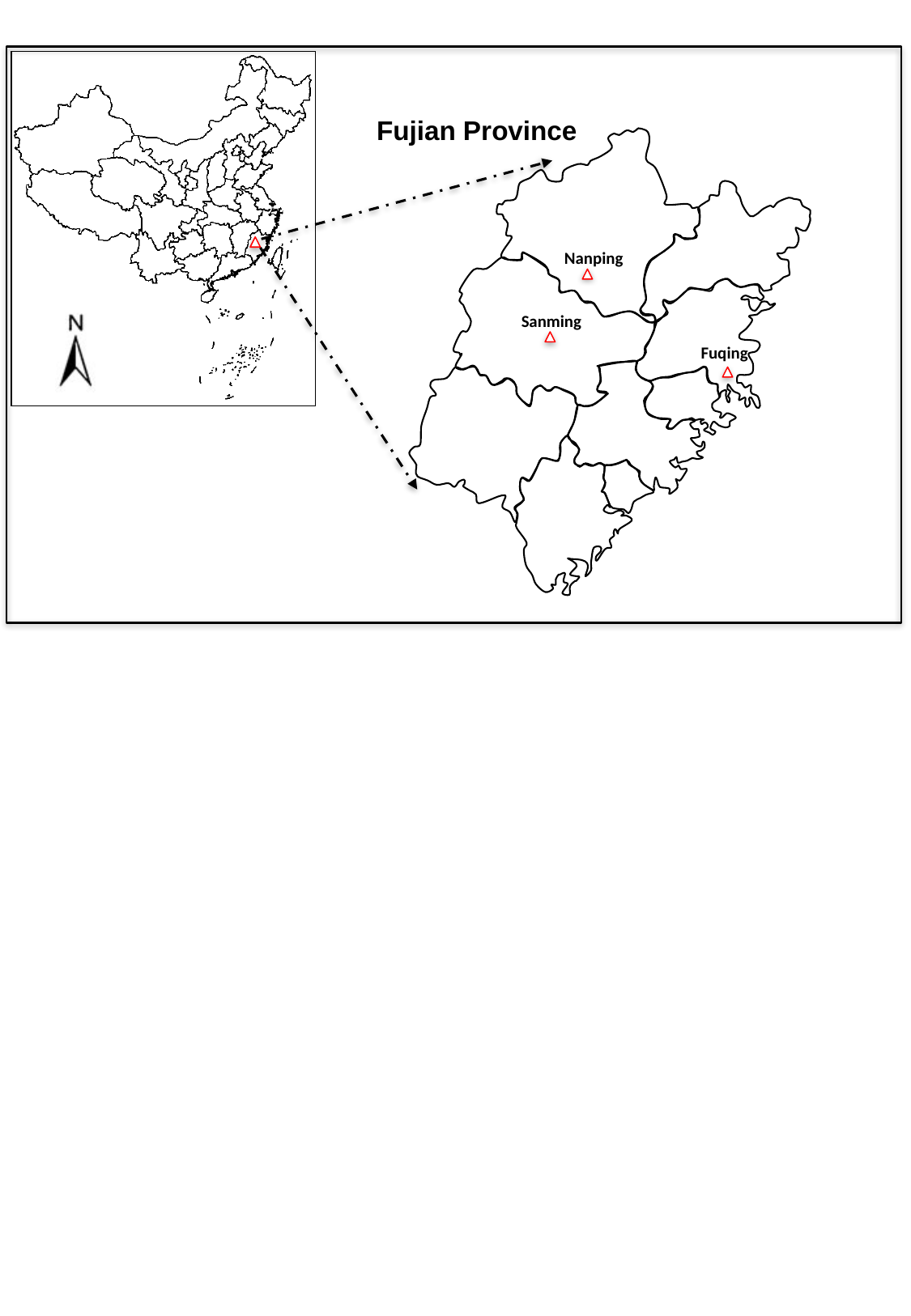

Fujian Province
Nanping
Sanming
Fuqing

Supplement: Supplementary file 1 [file viruses-15-01822-s001.zip › viruses-2551982-supplementary.pptx]
